# Immune-Related lncRNA Signature for Predicting the Immune Landscape of Head and Neck Squamous Cell Carcinoma

**DOI:** 10.3389/fmolb.2021.689224

**Published:** 2021-07-13

**Authors:** Ji Yin, Xiaohui Li, Caifeng Lv, Xian He, Xiaoqin Luo, Sen Li, Wenjian Hu

**Affiliations:** ^1^ Department of Otorhinolaryngology, Affiliated Traditional Chinese Medicine Hospital of Southwest Medical University, Luzhou, China; ^2^ Spinal Surgery Department, Affiliated Traditional Chinese Medicine Hospital of Southwest Medical University, Luzhou, China

**Keywords:** head and neck squamous cell carcinoma, long non-coding RNAs, immune, immunotherapy, prognostic model, bioinformation analysis

## Abstract

**Background:** Long non-coding RNA (lncRNA) plays a significant role in the development, establishment, and progression of head and neck squamous cell carcinoma (HNSCC). This article aims to develop an immune-related lncRNA (irlncRNA) model, regardless of expression levels, for risk assessment and prognosis prediction in HNSCC patients.

**Methods:** We obtained clinical data and corresponding full transcriptome expression of HNSCC patients from TCGA, downloaded GTF files to distinguish lncRNAs from Ensembl, discerned irlncRNAs based on co-expression analysis, distinguished differentially expressed irlncRNAs (DEirlncRNAs), and paired these DEirlncRNAs. Univariate Cox regression analysis, LASSO regression analysis, and stepwise multivariate Cox regression analysis were then performed to screen lncRNA pairs, calculate the risk coefficient, and establish a prognosis model. Finally, the predictive power of this model was validated through the AUC and the ROC curves, and the AIC values of each point on the five-year ROC curve were calculated to select the maximum inflection point, which was applied as a cut-off point to divide patients into low- or high-risk groups. Based on this methodology, we were able to more effectively differentiate between these groups in terms of survival, clinico-pathological characteristics, tumor immune infiltrating status, chemotherapeutics sensitivity, and immunosuppressive molecules.

**Results:** A 13-irlncRNA-pair signature was built, and the ROC analysis demonstrated high sensitivity and specificity of this signature for survival prediction. The Kaplan–Meier analysis indicated that the high-risk group had a significantly shorter survival rate than the low-risk group, and the chi-squared test certified that the signature was highly related to survival status, clinical stage, T stage, and N stage. Additionally, the signature was further proven to be an independent prognostic risk factor *via* the Cox regression analyses, and immune infiltrating analyses showed that the high-risk group had significant negative relationships with various immune infiltrations. Finally, the chemotherapeutics sensitivity and the expression level of molecular markers were also significantly different between high- and low-risk groups.

**Conclusion:** The signature established by paring irlncRNAs, with regard to specific expression levels, can be utilized for survival prediction and to guide clinical therapy in HNSCC.

## Introduction

Worldwide, head and neck cancers have yearly incidences of 930,000 cases and 470,000 deaths, involving malignant tumors in the lip, oral cavity, nasopharynx, oropharynx, hypopharynx, and larynx (Sung et al., 2021). Head and neck squamous cell carcinoma (HNSCC) accounts for more than 90% of all head and neck cancers, with the main risk factors for HNSCC being tobacco, alcohol, and human papillomavirus infection. Currently, surgery, radiotherapy, and chemotherapy are the main treatment strategies for HNSCC (Leemans et al., 2018). Over the past few years, large cohort clinical trials have demonstrated that immunotherapy plays a significant role in the treatment of HNSCC, especially for cases involving immune checkpoint inhibitors (ICIs) (Cramer et al., 2019).

The evolution and progression of tumors rely on the acquisition of traits that enable cancer cells to escape immune surveillance and an effective immune response. Therefore, cancer immunotherapy works on the premise that the host immune system can be sensitized to malignant cancer cells and be activated to attack them. ICIs are promising novel agents for malignancies, which work by blocking inhibitory immune checkpoint pathways to reactivate immune responses against cancer, such as anti-programmed death-1 (anti-PD-1), anti-programmed death-1 ligand (anti-PD-L1), and anti-cytotoxic T-lymphocyte-associated protein 4 (anti-CTLA-4) antibodies (Ferris, 2015). Pembrolizumab is an approved second-line option for the treatment of platinum-refractory recurrent/metastatic diseases, which is supported by KEYNOTE-040, indicating that pembrolizumab provided clinically significant benefits, with a median OS of 8.4 months for pembrolizumab *vs.* 6.9 months for standard therapy (Ho and Mehra, 2019). Additionally, the safety and feasibility of nivolumab are being studied for patients with resectable HNSCC in the CheckMate 358 trial (Pol, 2018).

Long non-coding RNAs (lncRNAs) are the most common RNA species, with the length exceeding 200bp (Li and Chen, 2016). Abundant evidence has indicated that lncRNAs are active participants in various stages of tumor immunity (Wu et al., 2020). Emerging evidence has suggested that lncRNAs as regulators have an important effect on cancer immunity, such as antigen release, antigen presentation, immune activation, immune cell migration, immune cell infiltration, and killing cancer cells (Denaro et al., 2019). Ji et al. reported that LNC-TIM3 can promote T cell exhaustion, which may be related to anti-tumor immunity (Ji et al., 2018). Huang et al. revealed that lncRNA TCONS_00019715 can advance macrophage polarization to the M1 phenotype and enhance antitumor activities (Huang et al., 2016).

Therefore, evaluating immune-related lncRNAs (irlncRNAs) can potentially be exploited for promising prognostic and predictive information associated with patient outcomes. Wei et al. constructed a nine-irlncRNA signature to evaluate the prognosis of patients with pancreatic cancer (Wei et al., 2019). Wu et al. integrated lncRNAs, microRNAs, and messenger RNAs related to the clinical data and set up a novel immune-related RNA signature to predict the survival of patients with HNSCC (Wu et al., 2019). Zhang et al. identified ten irlncRNAs and established a model to predict the survival of patients with hepatocellular carcinoma (Zhang et al., 2020).

By comparison, the combination of two biomarkers is superior to one biomarker in tumor diagnosis and prognosis prediction (Lv et al., 2019). A novel irlncRNA signature was constructed through a fresh modeling algorithm, paring, and iteration, which was not affected by the level of expression (Hong et al., 2020). The article aimed to establish a prognosis model based on the novel irlncRNA signatures for their predictive value, diagnostic effectiveness, chemotherapeutic efficacy, and tumor immune infiltration.

## Materials and Methods

### Data Preparation and Differentially Expressed irlncRNA Analysis

The RNA-seq data harmonized to fragments per kilobase million and corresponding clinical information of HNSCC were downloaded from The Cancer Genome Atlas (TCGA, https://tcga-data.nci.nih.gov/tcga/). The relevant data were extracted by eliminating duplicate data and data with a 0-day follow-up time. After adding annotation based on GTF files from the Ensembl database (http://asia.ensembl.org), the expression profiles of lncRNAs were obtained. A dataset of identified immune-related genes was acquired from the ImmPort database (http://www.immport.org) and was utilized to identify irlncRNAs through a co-expression strategy. Those lncRNAs were defined as irlncRNAs (cor > 0.4 and *p* value < 0.001). Finally, the R package limma was utilized to distinguish differentially expressed irlncRNA (DEirlncRNA) from irlncRNAs, and the threshold values were log fold-change (FC) > 1.0 and false discovery rate (FDR) < 0.05.

### Pairing DEirlncRNAs

The DEirlncRNAs were cyclically individually paired, and a 0-or-1 matrix was constructed such that C was equal to lncRNA A plus lncRNA B; if the expression level of lncRNA A was higher than that of lncRNA B, C was defined as 1; otherwise, C was defined as 0. Because some pairs without a certain rank could not correctly predict patient survival outcomes, whenever the amounts of lncRNA pairs whose expression quantity was 0 or 1 accounted for more than 20% and less than 80% of total pairs, it was considered a valid match.

### Establishment of Risk Model for Evaluating Risk Score

Univariate Cox regression analysis was utilized to evaluate the association of each valid lncRNA pair with survival, and lncRNA pairs with *p* < 0.05 were identified as candidate lncRNA pairs. Least absolute shrinkage and selection operator (LASSO) regression analysis was used to screen these candidate lncRNA pairs. Finally, the other candidate lncRNA pairs were further analyzed by stepwise multivariate Cox regression analysis to select a group of lncRNA pairs, thereby establishing a risk model. The risk score formula was as follows: . The specific risk score for each patient in the model was calculated.

To assess the predictive ability of the model for survival, the time-dependent receiver-operating characteristic (ROC) curves and the area under the curve (AUC) were evaluated. The Akaike information criterion (AIC) values of each point on the five-year ROC curve were calculated to select the maximum inflection point, which was then applied as a cut-off point to divide patients into low- or high-risk groups. The R packages used in these steps included limma, survival, survminer, glmnet, and survivalROC.

### Evaluation of the Prediction Model

To verify the accuracy of the cut-off point, we performed Kaplan–Meier analysis to show the difference in survival between high- and low-risk groups; the survival curve was then graphed. Next, the chi-squared test was applied to evaluate the relationship between the model and clinical characteristics. We then applied the Wilcoxon signed-rank test to calculate the risk score differences among different groups with these clinical characteristics. To confirm that the risk score was an independent predictor of clinical prognosis, univariate and multivariate Cox analyses were utilized to compare the relationship between the risk score and clinical characteristics and survival. These procedures were performed using R packages including survival, survminer, limma, ggpubr, and ComplexHeatmap.

### Exploration of Immune Cell Infiltration

In order to estimate reliable immune cell infiltration, we considered the commonly accepted methods for calculating the immune infiltration statuses in samples, including XCELL, TIMER, QUANTISEQ, MCPCOUNTER, EPIC, CIBERSORT abs, and CIBERSORT. The differences in immune infiltrating cell content between high- and low-risk groups of the model were analyzed by the Wilcoxon signed-rank test. We calculated the relationship between the risk score and immune infiltrating cells using the Spearman correlation analysis. The significance threshold was set to *p* < 0.05. The R packages used in these operations were limma, scales, ggplot2, ggtext, and ggpubr.

### Investigation of the Expression of ICI-Related Molecules

We carried out limma and ggpubr packages to study the relationship between the model and the expression level of ICI-related molecules.

### Analysis of Chemotherapeutic Drug Sensitivity

To evaluate the sensitivity of HNSCC chemotherapy in the model, we calculated the half inhibitory concentration (IC50) of commonly administrated chemotherapeutic drugs. The Wilcoxon signed-rank test was used to compare the difference in the IC50 between high- and low-risk groups. The above steps were performed through pRRophetic and ggplot2 R packages.

## Results

### Identification of DEirlncRNAs

**FIGURE 1:**
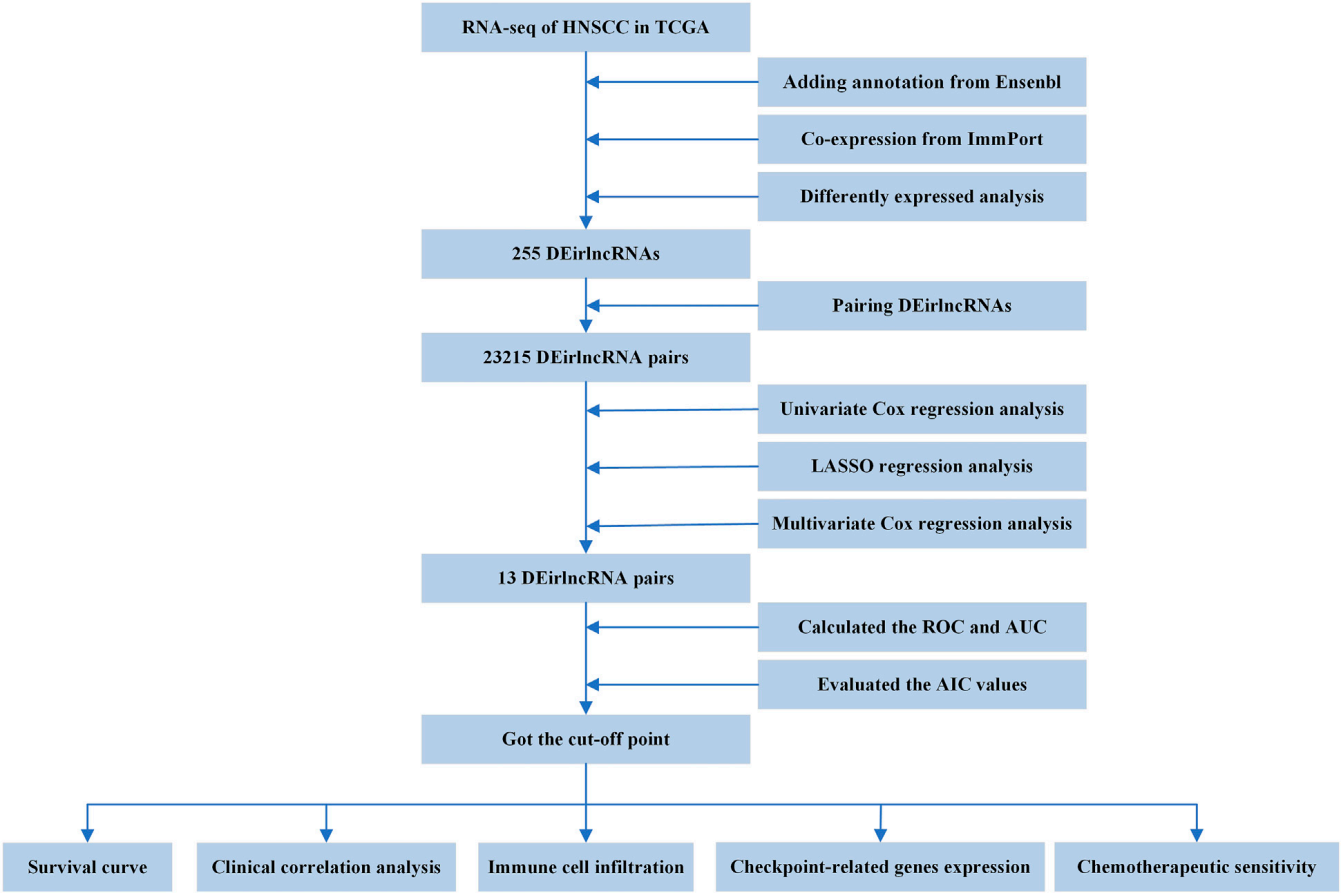
Flowchart of this article.

**FIGURE 2:**
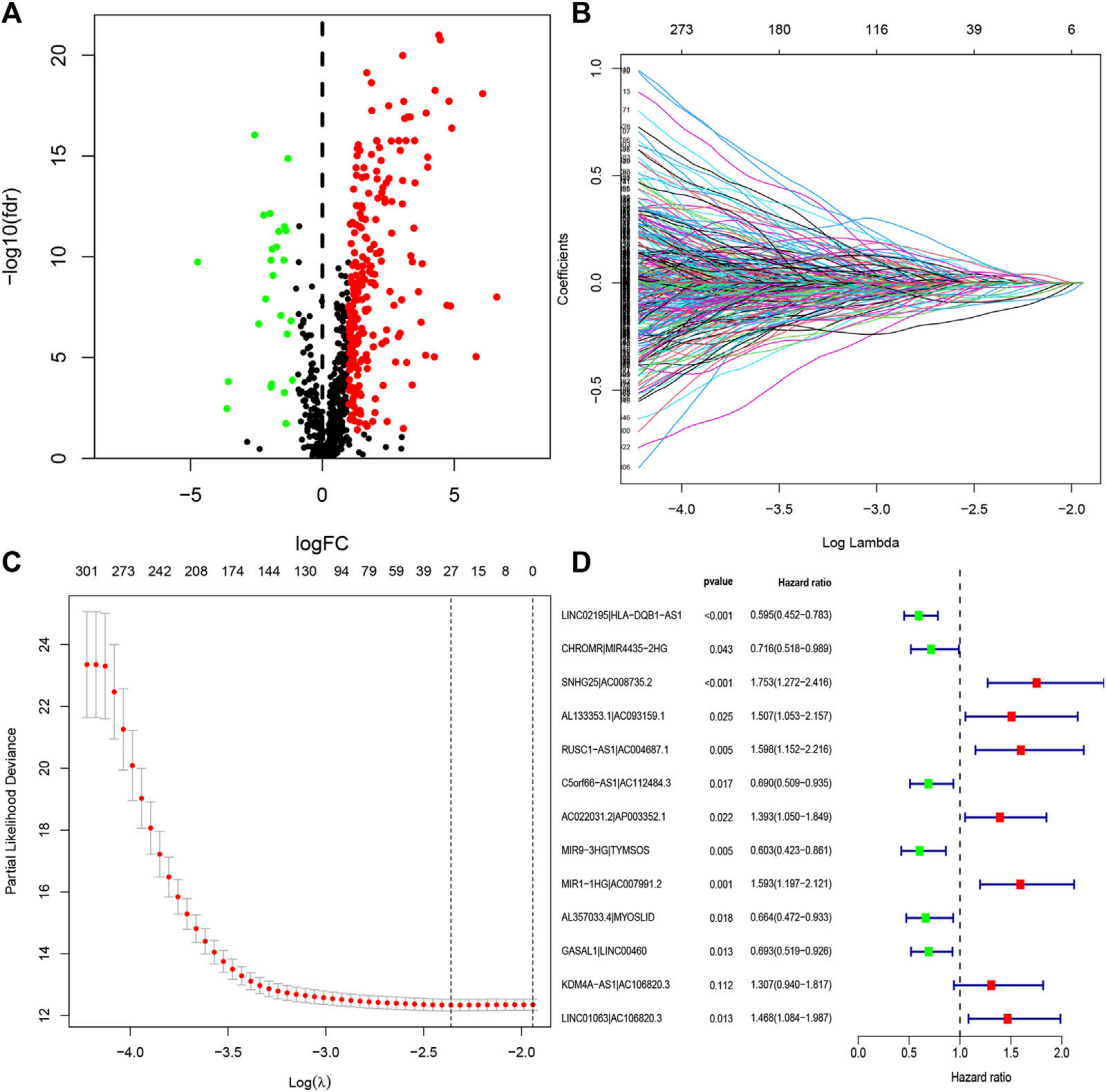
Identification of DEirlncRNAs and establishment of a risk assessment model. **(A)** Volcano diagram of DEirlncRNAs. The green, red, and black dots represent the down- and upregulated DEirlncRNAs and lncRNA that did not satisfy the screening criteria, respectively. **(B)** LASSO coefficient profiles of the 27 DEirlncRNA pairs. **(C)** A coefficient profile plot was generated against the log sequence. Selection of the optimal parameter in the LASSO model. **(D)** A forest map displaying 13 DEirlncRNA pairs identified by the Cox proportional hazards regression model for multivariate analyses using a forward stepwise method.

The entire process flowchart of this study is shown in Figure 1. First, the RNA-seq data and corresponding clinical information for HNSCC were obtained from TCGA, including 44 normal and 501 tumor samples. Next, the relationship was calculated based on co-expression analysis between lncRNAs and immune-related genes. Finally, a total of 722 irlncRNAs were extracted (Supplementary Table S1), and 255 irlncRNAs were distinguished into DEirlncRNAs, including 230 that were upregulated and 25 that were downregulated (Figure 2A).

### Construction and Evaluation of Risk Assessment Model

Through iterative loop and 0-or-1 matrix screening, 23,215 DEirlncRNA pairs were obtained from 255 DEirlncRNAs. A univariate Cox regression analysis was then performed to identify characteristics for prognostic prediction of patients, and 4,146 DEirlncRNA pairs were determined to be statistically significant (*p* < 0.05; Supplementary Table S2). Next, the LASSO regression analysis was further utilized for these 4,146 DEirlncRNA pairs; the corresponding LASSO coefficient profiles and a partial likelihood deviation plot are shown in Figures 2B,C, which reveal the 27 candidate DEirlncRNA pairs. Finally, multivariate Cox regression analysis by the stepwise method was applied, and 13 DEirlncRNA pairs were identified to establish the prognostic model in HNSCC (Figure 2D).

**FIGURE 3:**
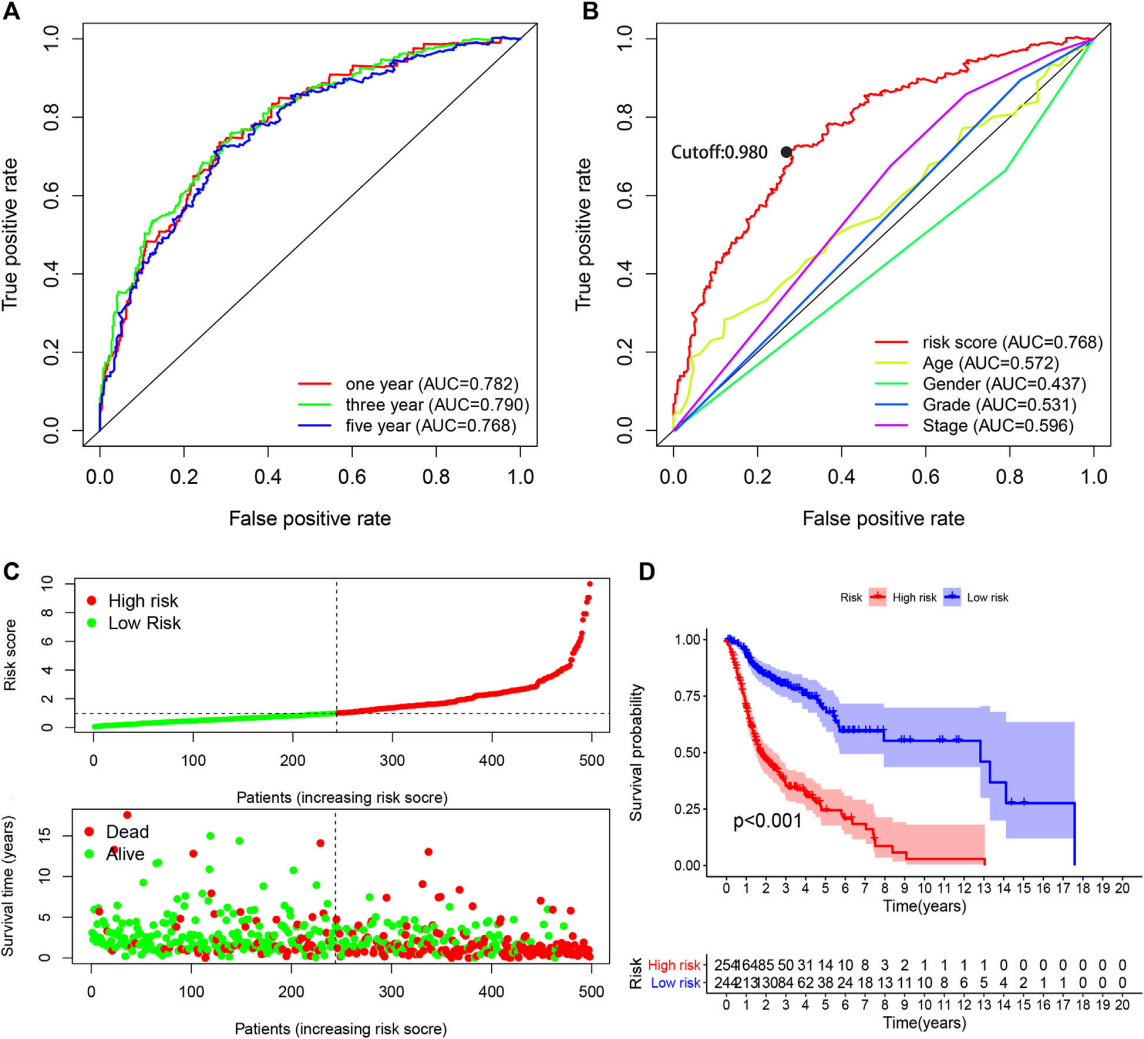
Performance evaluation of the risk model for survival prediction. **(A)** One-, three-, and five-year ROC curves of the optimal model indicating that all AUC values were more than 0.75. **(B)** A comparison of 5-year ROC curves with other clinical characteristics illustrating the superiority of this model. The maximum inflection point was the cut-off point obtained by the AIC. **(C)** Risk scores and survival outcome of each case. **(D)** The survival time of the high-risk group was shorter based on Kaplan–Meier analysis.

The AUC values were more than 0.75 in one-, three-, and five-year ROC curves in the model, suggesting high sensitivity and specificity of the 13-DEirlncRNA-pair signature for survival prediction (Figure 3A). The AUC of the ROC analysis for five-year survival prediction indicated that the risk score was highest at 0.768, which demonstrated that it was more accurate than other traditional clinical parameters (Figure 3B). Subsequently, we counted all the AIC values of the five-year ROC curve to identify the maximum inflection point as a cut-off point and utilized the cut-off point to distinguish high- and low-risk groups in the model (Figure 3B).

### Clinical Evaluation by Risk Assessment Model

**FIGURE 4:**
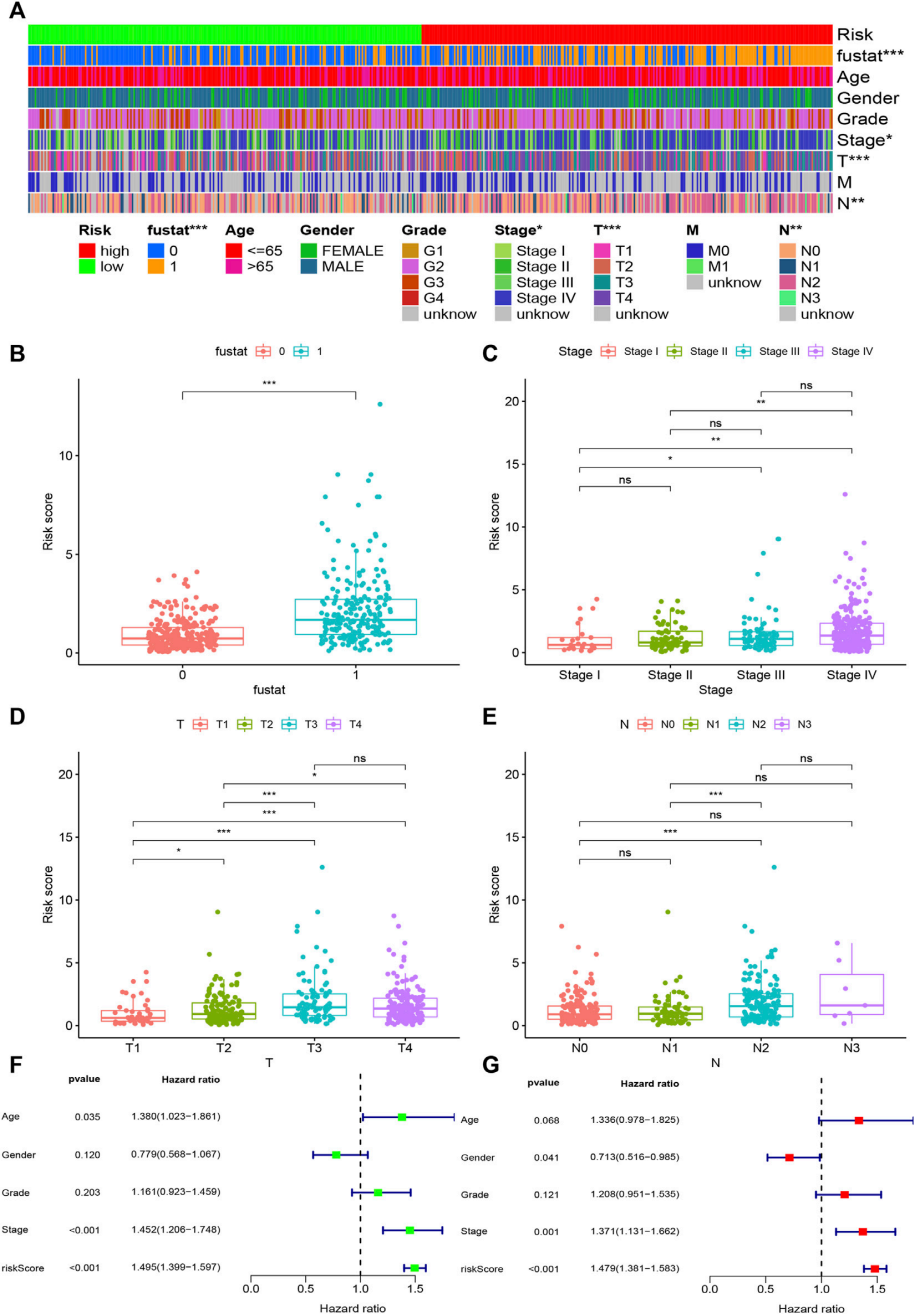
Evaluation of clinical characteristics by the risk assessment model. **(A–E)** A strip chart along with the scatter diagram shows that survival status, clinical stage, T stage, and N stage were significantly associated with the risk score. **(F, G)** Univariate Cox regression analysis shows that the risk score, age, and clinical stage were statistically different, while the corresponding multivariate Cox regression analysis shows that only the risk score and clinical stage were independent prognostic risk factors.

Based on the cut-off value (0.980), 254 patients were classified into the high-risk group and the remaining 244 patients into the low-risk group. The distribution of risk score for each HNSCC case is displayed in Figure 3C, which demonstrates that patients in the low-risk group generally had a better clinical outcome than those in the high-risk group. In addition, Kaplan–Meier analysis indicated that the high-risk HNSCC patients had a significantly shorter survival than the low-risk patients (*p* < 0.001; Figure 3D). A series of chi-squared tests were conducted to investigate the association of the risk score of HNSCC with clinico-pathological characteristics. The strip chart (Figure 4A) and consequent scatter diagrams demonstrate that the risk score was highly correlated with survival status (Figure 4B), clinical stage (Figure 4C), T stage (Figure 4D), and N stage (Figure 4E) of HNSCC patients. Next, univariate Cox regression analysis revealed that clinical factors, such as the risk score [HR = 1.495, 95% CI (1.399–1.597), *p* value <0.001], age [HR = 1.380, 95% CI (1.023–1.861), *p* value = 0.035], and clinical stage [HR = 1.452, 95% CI (1.206–1.748), *p* value <0.001], showed statistical differences (Figure 4F), but the corresponding multivariate Cox regression analysis showed that only the risk score [HR = 1.479, 95% CI (1.381–1.583), *p* value <0.001] and clinical stage [HR = 1.371, 95% CI (1.131–1.662), *p* value = 0.001] were independent prognostic risk factors (Figure 4G). The details of univariate and multivariate Cox regression analyses are presented in Supplementary Table S3.

### Evaluation of Tumor-Infiltrating Immune Cells and Immunosuppressive Molecules

**FIGURE 5:**
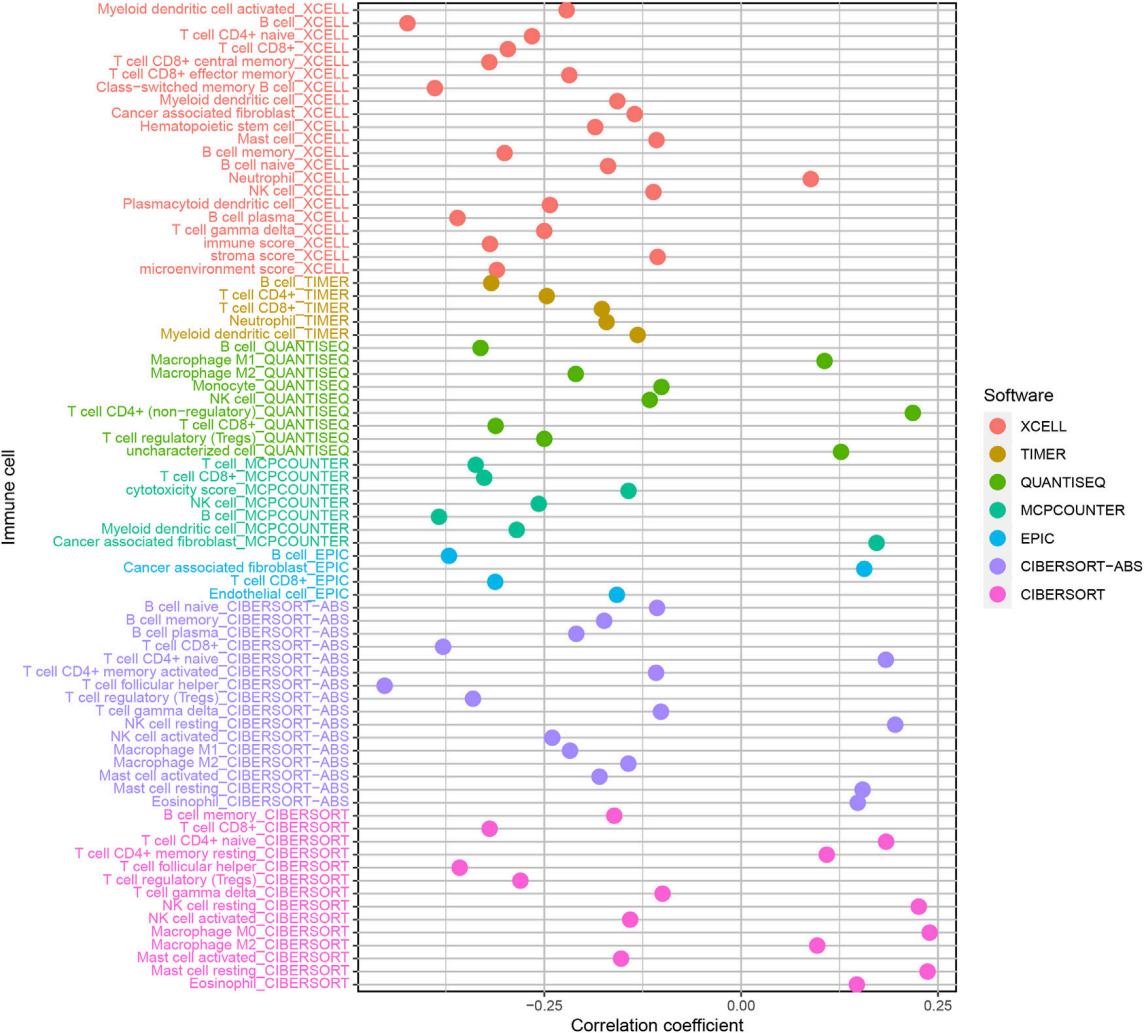
Estimation of tumor-infiltrating cells. The high-risk group in the model had significant negative relationships with immune infiltration of B cells, mast cells, myeloid dendritic cells, NK cells, regulatory T cells, CD4^+^ T cells, and CD8^+^ T cells.

**FIGURE 6:**
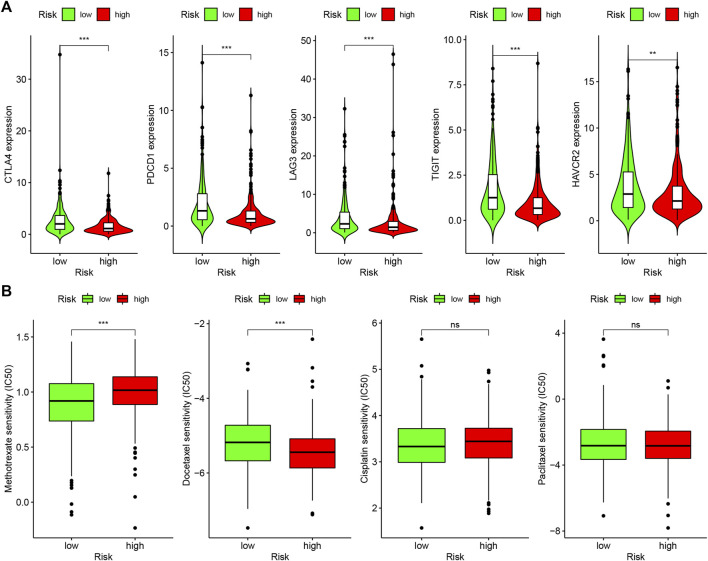
Differences of checkpoint-related gene expression and chemotherapeutic sensitivity in high- and low-risk groups. **(A)** The high-risk group in the model was negatively associated with CTLA-4, PDCD1, LAG3, TIGIT, and HAVCR2. **(B)** The high-risk group in the model was related to lower IC50 of chemotherapeutics of methotrexate, and the low-risk group was related to lower IC50 of chemotherapeutics of docetaxel, whereas, in high- and low-risk groups, there were no statistical differences for cisplatin and caclitaxel.

Because lncRNAs and immune-related genes were interrelated at the beginning, we further explored the relationship between the model and the tumor immune microenvironment. The results indicated that the high-risk group in the model had significant negative relationships with immune infiltration of B cells, mast cells, myeloid dendritic cells, NK cells, regulatory T cells, CD4^+^ T cells, and CD8^+^ T cells (Supplementary Figure S1). The detailed Spearman correlation analysis, as shown in Figure 5, is presented in Supplementary Table S4. We further investigated whether the model was related to ICIs and discovered that the high-risk group in the model was negatively associated with various immune checkpoint molecules (Figure 6A), such as CTLA-4 (*p* < 0.001), PDCD1 (*p* < 0.001), LAG3 (*p* < 0.001), TIGIT (*p* < 0.001), and HAVCR2 (*p* < 0.01).

### Correlation Analysis of Risk Model and Chemotherapeutics

In addition to checkpoint blocking therapy, we also attempted to explore the relationship between the model and the sensitivity of common chemotherapeutics in treating HNSCC. We discovered that the high-risk group in the model was correlated with lower IC50 of chemotherapeutics of methotrexate (*p* < 0.001) and the low-risk group was correlated with lower IC50 of chemotherapeutics of docetaxel (*p* < 0.001); however, there was no statistical difference between cisplatin and caclitaxel in high- and low-risk groups, which demonstrates that the model can be utilized as a potential predictor of chemotherapeutics sensitivity (Figure 6B).

## Discussion

An increasing number of studies have focused on establishing irlncRNA signatures to evaluate the prognosis of patients with malignant tumors (Li T. et al., 2020). However, due to the inherent biological heterogeneity of cancers and technical bias, traditional prognostic models require the standardization of expression profiles, making data analysis more complex. Instead, the two lncRNA combinations based on the relative ranking of gene expression levels can be used without the need for standardization (Peng et al., 2018; Meng et al., 2020). In this article, we have attempted to construct a reasonable model with two lncRNA combinations, which are not affected by the level of expression.

Firstly, we obtained the RNA-seq data from TCGA, utilized differential co-expression analysis to discriminate DEirlncRNAs, and extracted DEirlncRNA pairs by iterative loop and 0-or-1 matrix screening. Secondly, we screened these DEirlncRNA pairs by univariable Cox regression analysis, LASSO regression analysis, and multivariate Cox regression analysis and constructed a prognostic model. Thirdly, we not only established an optimal model but also calculated the AIC values of each point on the ROC curve to determine the optimal cut-off point to distinguish high- and low-risk groups in HNSCC patients. Finally, we evaluated this novel model from the viewpoints of survival, clinic pathological characteristics, infiltrating immune cells, immune checkpoint molecules, and chemotherapeutics sensitivity.

Many studies have shown that various lncRNAs are related to the prognosis of patients with a variety of malignant tumors, especially for HNSCC. Wang et al. analyzed lncRNA expression profiles of HNSCC patients from TCGA to identify a three-lncRNA signature that could predict the survival of patients with HNSCC (Wang et al., 2018). Liu et al. constructed a prognostic risk model with five lncRNAs to predict the prognostic differences of HNSCC patients; this model can also be utilized in postoperative treatment and follow-up (Liu et al., 2018). Previous studies have found that various lncRNAs that have been identified in the modeling process play an important role in a variety of malignant tumors including HNSCC. Xie et al. reported that LINC00460 promotes HNSCC progression by sponging miR-612 to upregulate AKT2 (Xie et al., 2019). Xiong et al. revealed that MYOSLID accelerates invasion and metastasis by regulating the epithelial–mesenchymal transition progress in HNSCC (Xiong et al., 2019). Increasing evidence has suggested that lncRNAs play fundamental roles in cancer immune processes, such as the immune microenvironment and the activation of immune cells. Wu et al. studied irlncRNAs in the TCGA HNSCC project and validated them in GSE65858 from the GEO database (Wu et al., 2019). Therefore, we believe that the model we developed during our research also has the potential to determine new biomarkers for further study.

An increasing number of studies have shown that tumor immune cell infiltration is significantly related to the sensitivity to ICIs. For example, a clinical trial indicated that patients with high levels of CD8^+^ T cell infiltration experienced a superior treatment response from pembrolizumab (Garon et al., 2019). To make reliable immune infiltration estimations, seven common acceptable methods, including XCELL, TIMER, QUANTISEQ, MCPCOUNTER, EPIC, CIBERSORT abs, and CIBERSORT, were utilized to estimate immune infiltrating cells (Becht et al., 2016; Aran et al., 2017; Racle et al., 2017; Chen et al., 2018; Li M. et al., 2020; Plattner et al., 2020). By integrating and analyzing these results, we discovered that the high-risk group in our model had significant negative relationships with immune infiltration of B cells, mast cells, myeloid dendritic cells, NK cells, regulatory T cells, CD4^+^ T cells, and CD8^+^ T cells. Currently, the role of B cells has been controversial: some studies showed that B cell infiltration in tumors was associated with poor prognosis, while others indicated the opposite (Tsou et al., 2016; Sarvaria et al., 2017). Similarly, regulatory T cells were negatively associated with the prognosis of patients in some studies, but other researchers have found that regulatory T cells may inhibit malignant transformation in special situations of tumor initiation (Wolf et al., 2015). Our results showed that the low-risk group in the model was positively associated with CTLA-4, PDCD1, LAG3, TIGIT, and HAVCR2 immune checkpoint molecules, and the IC50 of these drugs in the high-risk group was statistically different, such as with methotrexate and docetaxel.

It is important to acknowledge that the current study has a number of shortcomings and limitations. First, the model would be more convincing if it could be verified with external data. Second, in TCGA HNSCC datasets, since most of the patients are non-metastatic, the results may be biased. Third, our total sample size (44 normal and 501 tumor samples) is relatively small. Therefore, we plan to collect additional clinical samples and expand the sample size for further examination and confirmation of our model.

Taken together, our study identified a novel signature that did not require specific lncRNA expression levels, which could predict the prognosis for patients with HNSCC and might present valuable clinical applications in antitumor immunotherapy.

## Data Availability

The datasets presented in this study can be found in online repositories. The names of the repository/repositories and accession number(s) can be found in the article/Supplementary Material.
