# Supplementary figures and images for: Immune-Related lncRNA Signature for Predicting the Immune Landscape of Head and Neck Squamous Cell Carcinoma
Source: Front Mol Biosci. 2021 Jul 13;8:689224. doi: 10.3389/fmolb.2021.689224 (PMC8313825; doi:10.3389/fmolb.2021.689224)

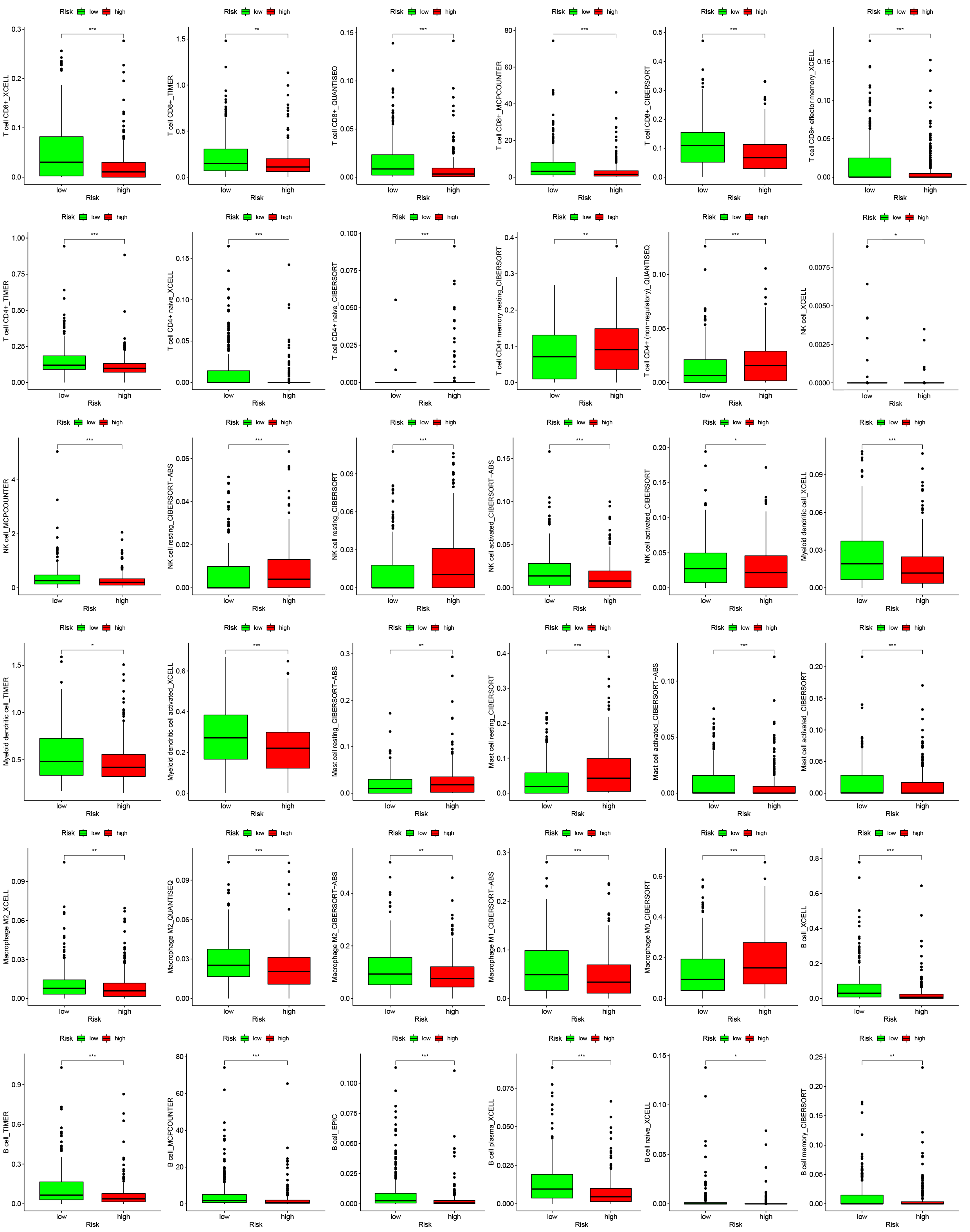

Supplement: Supplementary file 3 [file Image1.TIF]
