# Supplementary material for: Immune-Related lncRNA Signature for Predicting the Immune Landscape of Head and Neck Squamous Cell Carcinoma
Source: Front Mol Biosci. 2021 Jul 13;8:689224. doi: 10.3389/fmolb.2021.689224 (PMC8313825; doi:10.3389/fmolb.2021.689224)
Supplement: Supplementary file 4 [file Table3.DOCX]

| Univariate Cox regression analysis | | | | |
| --- | --- | --- | --- | --- |
| id | HR | HR.95L | HR.95H | pvalue |
| Age | 1.379535674 | 1.022716648 | 1.860846481 | 0.03511178 |
| Gender | 0.778807585 | 0.568422954 | 1.06705975 | 0.119713964 |
| Grade | 1.160543194 | 0.922834925 | 1.459481505 | 0.20293652 |
| Stage | 1.451799835 | 1.205963064 | 1.747750677 | 8.20E-05 |
| riskScore | 1.494999552 | 1.399113918 | 1.597456527 | 1.33349E-32 |
| Multivariate Cox regression analysis | | | | |
| id | HR | HR.95L | HR.95H | pvalue |
| Age | 1.336162888 | 0.978318919 | 1.824897002 | 0.06843319 |
| Gender | 0.712812389 | 0.515591541 | 0.985472919 | 0.04050974 |
| Grade | 1.208469742 | 0.951429874 | 1.534951925 | 0.120685252 |
| Stage | 1.370946241 | 1.130791737 | 1.662104111 | 0.001323063 |
| riskScore | 1.478587553 | 1.381273818 | 1.582757251 | 2.09E-29 |
