# Supplementary material for: Immune-Related lncRNA Signature for Predicting the Immune Landscape of Head and Neck Squamous Cell Carcinoma
Source: Front Mol Biosci. 2021 Jul 13;8:689224. doi: 10.3389/fmolb.2021.689224 (PMC8313825; doi:10.3389/fmolb.2021.689224)
Supplement: Supplementary file 5 [file Table4.DOCX]

| immune | cor | pvalue |
| --- | --- | --- |
| B cell_TIMER | -0.31739925 | 4.06E-13 |
| T cell CD4+_TIMER | -0.246919268 | 2.36E-08 |
| T cell CD8+_TIMER | -0.176889096 | 7.22E-05 |
| Neutrophil_TIMER | -0.170942319 | 0.000126385 |
| Myeloid dendritic cell_TIMER | -0.131379672 | 0.003311574 |
| B cell memory_CIBERSORT | -0.161178465 | 0.000304495 |
| T cell CD8+_CIBERSORT | -0.319439519 | 2.82E-13 |
| T cell CD4+ naive_CIBERSORT | 0.184107407 | 3.57E-05 |
| T cell CD4+ memory resting_CIBERSORT | 0.108739117 | 0.015194179 |
| T cell follicular helper_CIBERSORT | -0.35744064 | 1.87E-16 |
| T cell regulatory (Tregs)_CIBERSORT | -0.280383505 | 1.90E-10 |
| T cell gamma delta_CIBERSORT | -0.099839107 | 0.025882433 |
| NK cell resting_CIBERSORT | 0.225645627 | 3.61E-07 |
| NK cell activated_CIBERSORT | -0.14094263 | 0.001615392 |
| Macrophage M0_CIBERSORT | 0.239479503 | 6.31E-08 |
| Macrophage M2_CIBERSORT | 0.096641806 | 0.031061808 |
| Mast cell activated_CIBERSORT | -0.152504316 | 0.000638487 |
| Mast cell resting_CIBERSORT | 0.236788397 | 8.94E-08 |
| Eosinophil_CIBERSORT | 0.146956485 | 0.001005078 |
| B cell naive_CIBERSORT-ABS | -0.106798296 | 0.017119338 |
| B cell memory_CIBERSORT-ABS | -0.173974096 | 9.52E-05 |
| B cell plasma_CIBERSORT-ABS | -0.209394948 | 2.44E-06 |
| T cell CD8+_CIBERSORT-ABS | -0.378559583 | 2.05E-18 |
| T cell CD4+ naive_CIBERSORT-ABS | 0.183807239 | 3.68E-05 |
| T cell CD4+ memory activated_CIBERSORT-ABS | -0.108068968 | 0.015836279 |
| T cell follicular helper_CIBERSORT-ABS | -0.452873356 | 1.49E-26 |
| T cell regulatory (Tregs)_CIBERSORT-ABS | -0.340662212 | 5.36E-15 |
| T cell gamma delta_CIBERSORT-ABS | -0.101894055 | 0.022961996 |
| NK cell resting_CIBERSORT-ABS | 0.195716966 | 1.09E-05 |
| NK cell activated_CIBERSORT-ABS | -0.239918332 | 5.96E-08 |
| Macrophage M1_CIBERSORT-ABS | -0.217353497 | 9.74E-07 |
| Macrophage M2_CIBERSORT-ABS | -0.143265653 | 0.001347714 |
| Mast cell activated_CIBERSORT-ABS | -0.179907307 | 5.40E-05 |
| Mast cell resting_CIBERSORT-ABS | 0.154299042 | 0.000549506 |
| Eosinophil_CIBERSORT-ABS | 0.148122166 | 0.000914854 |
| B cell_QUANTISEQ | -0.331058128 | 3.35E-14 |
| Macrophage M1_QUANTISEQ | 0.105939321 | 0.018037374 |
| Macrophage M2_QUANTISEQ | -0.21005817 | 2.26E-06 |
| Monocyte_QUANTISEQ | -0.10121394 | 0.023895274 |
| NK cell_QUANTISEQ | -0.116101882 | 0.009509199 |
| T cell CD4+ (non-regulatory)_QUANTISEQ | 0.218079815 | 8.94E-07 |
| T cell CD8+_QUANTISEQ | -0.311807515 | 1.09E-12 |
| T cell regulatory (Tregs)_QUANTISEQ | -0.250018716 | 1.55E-08 |
| uncharacterized cell_QUANTISEQ | 0.126841189 | 0.004583682 |
| T cell_MCPCOUNTER | -0.337156494 | 1.05E-14 |
| T cell CD8+_MCPCOUNTER | -0.326372375 | 7.99E-14 |
| cytotoxicity score_MCPCOUNTER | -0.143025455 | 0.001373368 |
| NK cell_MCPCOUNTER | -0.257048553 | 5.89E-09 |
| B cell_MCPCOUNTER | -0.383745456 | 6.42E-19 |
| Myeloid dendritic cell_MCPCOUNTER | -0.285039794 | 9.18E-11 |
| Cancer associated fibroblast_MCPCOUNTER | 0.17203918 | 0.000114146 |
| Myeloid dendritic cell activated_XCELL | -0.221634363 | 5.86E-07 |
| B cell_XCELL | -0.423817725 | 3.97E-23 |
| T cell CD4+ naive_XCELL | -0.265677943 | 1.72E-09 |
| T cell CD8+_XCELL | -0.296285463 | 1.51E-11 |
| T cell CD8+ central memory_XCELL | -0.319797286 | 2.64E-13 |
| T cell CD8+ effector memory_XCELL | -0.218272394 | 8.74E-07 |
| Class-switched memory B cell_XCELL | -0.389028626 | 1.93E-19 |
| Myeloid dendritic cell_XCELL | -0.157215493 | 0.000429091 |
| Cancer associated fibroblast_XCELL | -0.135222779 | 0.002495085 |
| Hematopoietic stem cell_XCELL | -0.18534615 | 3.16E-05 |
| Mast cell_XCELL | -0.107492465 | 0.016407564 |
| B cell memory_XCELL | -0.300432631 | 7.57E-12 |
| B cell naive_XCELL | -0.169059324 | 0.000150313 |
| Neutrophil_XCELL | 0.088455279 | 0.048510547 |
| NK cell_XCELL | -0.111369323 | 0.01288967 |
| Plasmacytoid dendritic cell_XCELL | -0.242902537 | 4.03E-08 |
| B cell plasma_XCELL | -0.360276876 | 1.04E-16 |
| T cell gamma delta_XCELL | -0.250100017 | 1.54E-08 |
| immune score_XCELL | -0.319005718 | 3.04E-13 |
| stroma score_XCELL | -0.106075503 | 0.01788902 |
| microenvironment score_XCELL | -0.310250795 | 1.42E-12 |
| B cell_EPIC | -0.371098031 | 1.05E-17 |
| Cancer associated fibroblast_EPIC | 0.156395377 | 0.000460199 |
| T cell CD8+_EPIC | -0.31235576 | 9.88E-13 |
| Endothelial cell_EPIC | -0.157848288 | 0.000406438 |
